# Responder analyses for anti-amyloid immunotherapies for Alzheimer’s disease: a paradigm shift by regulatory authorities is urgently needed

**DOI:** 10.1093/braincomms/fcad276

**Published:** 2023-10-24

**Authors:** Simone Salemme, Antonio Ancidoni, Nicola Vanacore

**Affiliations:** Department of Biomedical, Metabolic, and Neural Sciences, University of Modena and Reggio Emilia, Modena 41125, Italy; National Center for Disease Prevention and Health Promotion, Italian National Institute of Health, Rome 00161, Italy; Department of Public Health and Infectious Diseases, Sapienza University, Rome 00185, Italy; National Center for Disease Prevention and Health Promotion, Italian National Institute of Health, Rome 00161, Italy

We read the article by Liu *et al*.^1^ with great interest and share several concerns of the authors regarding the risk–benefit ratio of anti-amyloid antibodies for the treatment of Alzheimer’s dementia.

We would specifically like to expand the discussion on the responder analyses using minimal clinically important difference (MCID) thresholds. The authors suggest that this type of analysis should be cautiously interpreted because (i) within-individual treatment responses cannot be assessed in parallel-arm trials; (ii) potentially serious statistical limitations are present as the consequence of a loss of statistical power; (iii) responder analyses are less effective at disentangling the treatment outcome from any true treatment effect; and (iv) dichotomization may lead to overestimating or underestimating borderline scenarios of continuous treatment effects.^1^ We agree with the list of these limits and the proposed approaches defined by the authors such as presenting a full tabulation of all statistical analyses, providing statistical details useful for the adjustment for multiple hypotheses testing and performing sensitivity analyses on possibly convergent results from functional and cognitive assessments. Adopting a public health perspective, it is our opinion that these topics should be further contextualized in the regulatory field. Statistical analyses using the MCID construct and/or patient-oriented outcomes (PRO) are specifically requested as desirable data by both the FDA and EMA.^2,3^ The FDA states that analyses evaluating what constitutes a meaningful within-patient change (i.e. improvement and deterioration from the patients’ perspective) as determined by clinical outcome assessments are of utmost relevance for regulatory decision-making.^2^ The EMA—together with the International Council for Harmonisation of Technical Requirements for Pharmaceuticals for Human Use (ICH)—proposed a guideline work to advance Patient Focused Drug Development with the aim to include PRO in the assessment of a drug.^3^ As for aducanumab, lecanemab and donanemab, the primary end-point (Clinical Dementia Rating Scale Sum of Boxes [CDR-SB] or integrated Alzheimer Disease Rating Scale [iADRS]) evaluation of phase 3 clinical trials consisted of statistical analyses of mean differences between groups.^3-5^ While statistically significant, the minimal mean differences observed are clinically questionable.^3-5^ Responder analyses using MCID thresholds or hard end-points can only be found in the supplementary analyses of EMERGE and ENGAGE (i.e. responder analyses adopting a delta ≤ 0.5 or 1.5 at the CDR-SB), among analyses of secondary efficacy end-points of Clarity AD (i.e. time to worsening of Global CDR Score) or among exploratory analyses of TRAILBLAZER-ALZ 2 (i.e. MCID thresholds for CDR-SB and iADRS and Global CDR score progression).^4-6^ Although some of the analyses present methodological or statistical issues (e.g. adopting a delta ≥ 0.5 at the CDR-SB and an intention-to-treatment approach would have been more appropriate), they provide useful data for appropriately evaluating these drugs.^4-6^ Unfortunately, such relevant data must be searched with difficulty among the numerous statistical analyses conducted in a trial and may become, depending on the situation, the subject of communication programmes in which the difference between exploratory and primary analyses blurs.^4-6^

In light of this, we wonder why statistical analyses adopting the MCID approach (i.e. responder analyses) or hard end-points (e.g. transition from Mild Cognitive Impairment (MCI) to dementia or time to worsening of Global CDR Score) are not formally required by the regulatory authorities as primary or co-primary analyses.

To our knowledge, only two studies have done so in patients with MCI or AD to date. The first Randomized Clinical Trial (RCT) was conducted on 769 amnestic MCI participants. The primary end-point was the time to diagnosis of possible or probable AD. Participants were randomly assigned to receive 2000 IU of vitamin E daily, 10 mg of donepezil daily or placebo for three years. Compared to the placebo group, no significant differences in the primary outcome were observed in the vitamin E group (HR = 1.02; 95% CI 0.74 to 1.41; *P* = 0.91) nor the donepezil group (HR = 0.80; 95% CI 0.57 to 1.13; *P* = 0.42).^7^ The second RCT was conducted on 565 community-resident participants with mild to moderate AD. The primary end-points were institutionalization and progression of disability. Patients were randomized to either donepezil (5 or 10 mg/day) or placebo for three years. Compared to placebo, no significant differences were observed in the donepezil group in institutionalization (relative risk = 0.97, 95% CI 0.72 to 1.30; *P* = 0.8) nor in progression of disability or institutionalization (relative risk = 0.96, 95% CI 0.74 to 1.24; *P* = 0.7).^8^ Regarding the validity of MCID thresholds, it has been criticized that MCID is based on arbitrary measures of clinical improvement. It is indeed our opinion that only MCID thresholds thoroughly validated by rigorous studies should be adopted in RCTs. In a recent prospective study performed on 451 cognitively unimpaired and 292 MCI participants, MCID thresholds for widely adopted cognitive tests have been proposed.^9^ In this context, it is commendable that USA funded the National Institute on Aging (NIA) ‘IM’bedded ‘P’ragmatic ‘A’lzheimer’s disease (AD) and AD-Related Dementias (AD/ADRD) ‘C’linical ‘T’rials (IMPACT) Collaboratory with the aim to conduct pragmatic trials for people living with dementia and their caregivers, and to develop adequate statistical methodology and guidance.^10^

We believe that a paradigm shift in the evaluation of drugs by the regulatory authorities is urgently needed, and this need is cross-cutting and interdisciplinary, reaching medical fields as a whole. For example, a systematic review was performed to evaluate the clinical meaningfulness of oncology therapies approved by the FDA and EMA according to American and European Society of Clinical Oncology definitions of quality of life.^11^ The authors showed that 6% of FDA- and 11% of EMA-approved indications met clinically meaningful benefits beyond MCID, concluding by stating that ‘*Of indications with evidence of statistical improvement, few have demonstrated clinically meaningful improvements*’.^11^ In conclusion, we propose that the new paradigm for research and approval of drugs for the treatment of dementia should be based on five criteria essential to increase the generalizability of trial data as follows: (i) the adoption of validated MCID thresholds or hard end-points as primary or co-primary analysis; (ii) the inclusion of at least one patient-reported outcome; (iii) the conduction of at least one pragmatic trial of interventions; (iv) the adoption of delayed start design if drugs are proposed as disease-modifying; and (v) the adoption of validated surrogate end-point. At last, we agree with Liu *et al.*^1^ that ‘*Sponsors should implement a data-sharing plan to make individual patient-level clinical trial data publicly available in a timely manner to qualified investigators, allowing external evaluation of study design and analyses*’.

## Data Availability

Data sharing is not applicable to this article as no new data were created or analysed in this study.
